# P-2230. Light Transmission-based Quantitation of Bacterial Growth on an Agar Plate

**DOI:** 10.1093/ofid/ofae631.2384

**Published:** 2025-01-29

**Authors:** Kyohei Umebayashi, Izumi Kawaguchi, Reiichi Ariizumi, Akihiko Fujisawa, Tomoya Tezen, Takanori Tsunashima, Kaoru Ito, Daichi Abe, Kazunori Yamaguchi, Masakazu Nakajima, Makoto Taketani, Kazune Matsumura

**Affiliations:** CarbGeM Inc., Kobe, Hyogo, Japan; CarbGeM Inc., Kobe, Hyogo, Japan; CarbGeM Inc., Kobe, Hyogo, Japan; Japan Display Inc., Ebina-shi, Kanagawa, Japan; Japan Display Inc., Ebina-shi, Kanagawa, Japan; Japan Display Inc., Ebina-shi, Kanagawa, Japan; Japan Display Inc., Ebina-shi, Kanagawa, Japan; Japan Display Inc., Ebina-shi, Kanagawa, Japan; Japan Display Inc., Ebina-shi, Kanagawa, Japan; CarbGeM Inc., Kobe, Hyogo, Japan; CarbGeM Inc., Kobe, Hyogo, Japan; Japan Display Inc., Ebina-shi, Kanagawa, Japan

## Abstract

**Background:**

The growth of microorganisms in liquid culture is usually evaluated by measuring light transmission and calculating optical density (OD). We have developed a new thin-film transistor image sensor (1000 x 1000 pixels) to measure light transmission through agar plates (Figure 1). Similar to OD in liquid culture, cells grown on the surface of agar plates scatter light and reduce the intensity of transmitted light as the cells grow. This device captures a single image every 5 minutes, enabling in-depth time-lapse studies of cell growth when combined with digital image analysis. Here we present proof-of-concept studies to apply this growth monitoring system for quantitative growth assessment.

**Figure 1.**
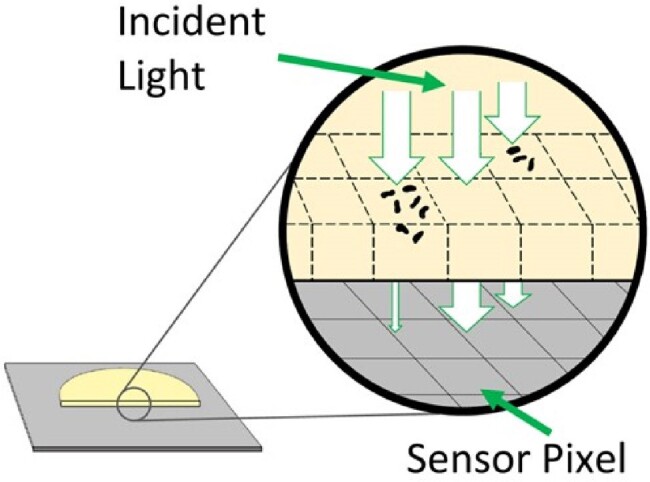
Schematic representation of the bacterial growth monitoring system.

**Methods:**

*Escherichia coli* cells were spread on a plate count agar plate and the growth was monitored over 745 min using the device. The optical density was calculated in the same way as OD of liquid culture: OD = -log_10_(I/I_0_) where I_0_ and I represent the intensity of incident light and transmitted light, respectively. The intensity of I_0_ and I was measured pixel by pixel; I_0_ was defined as the average of the first ten frames; I was defined as the average of the three consecutive frames (n-1, n, n+1) to reduce noise. The pixel size is 100 x 100 μm.

**Figure 2.**
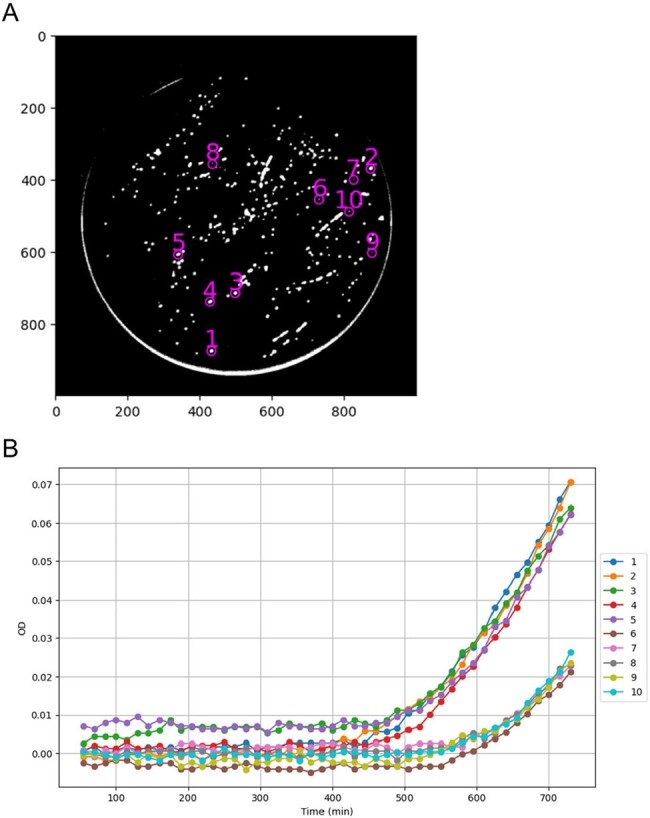
Quantitative evaluation of E. coli colony growth on an agar plate.

(A) The last (149th) frame was binarized at the threshold of OD 0.02 to extract colony contours. The circled and numbered colonies were analyzed in (B). The numbers along the horizontal and vertical axes represent the pixel positions. (B) The OD values of the central pixels of the colonies were tracked over time.

**Results:**

Colony contours were extracted from the binary image, at the threshold of OD 0.02, of the last (149^th^) frame (Figure 2A). Five colonies with the highest and lowest OD were selected and the OD values of their central pixels were plotted over time, showing distinct modes of increase (Figure 2B). Next, one colony (#3) was chosen to analyze whether the OD correlated with colony size. One axis represents the OD of its central pixel. The other represents the number of pixels that showed OD 0.02 or more in the 20 x 20 pixel area surrounding the central pixel. As shown in Figure 3, after approximately 550 min, the cell growing area began to expand in correlation with the continued increase in OD. The binarized images, at the indicated time points, also showed the area expansion.

Cell growth

**Figure:**
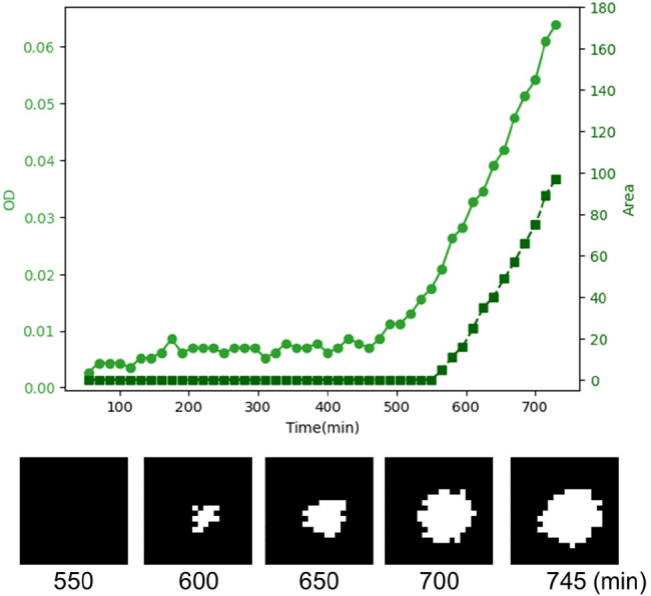

Figure 3. Cell growth in a single pixel and colony expansion in a wider area.

The colony #3 in Figure 2 was analyzed. Shown are the OD of the central pixel (light green) and the number of the pixels that showed OD 0.02 or more in the 20 x 20 pixel area surrounding the central pixel (dark green). The binarized images, at the indicated time points, also showed the area expansion.

**Conclusion:**

The light transmission-based growth monitoring system enabled us to quantitatively evaluate growth on the agar plate. The pixel-by-pixel analysis provided detailed information on the colony formation and growth. This system can be used in a broad spectrum of microorganism research and inspection.

**Disclosures:**

Masakazu Nakajima, B. Engineering, CarbGeM Inc.: Board Member|CarbGeM Inc.: Ownership Interest

